# Evaluation of radiofluorinated carboximidamides as potential IDO-targeted PET tracers for cancer imaging

**DOI:** 10.18632/oncotarget.14898

**Published:** 2017-01-30

**Authors:** Xuan Huang, Zhongjie Pan, Michael L Doligalski, Xia Xiao, Epifanio Ruiz, Mikalai M Budzevich, Haibin Tian

**Affiliations:** ^1^ Department of Cancer Imaging and Metabolism, H. Lee Moffitt Cancer Center & Research Institute, Tampa, FL, USA; ^2^ Department of Vascular Medicine, Tianjin Union Medicine Center, Tianjin, China; ^3^ Department of Pathology, Guangzhou University of Chinese Medicine, Guangzhou, China

**Keywords:** indoleamine 2, 3-dioxygenase (IDO), [^18^F]IDO49, PET, novel radioligand, immunotherapy

## Abstract

IDO1 is an enzyme catalyzing the initial and rate-limiting step in the catabolism of tryptophan along the kynurenine pathway. IDO1 expression could suppress immune responses by blocking T-lymphocyte proliferation locally, suggesting a role of IDO in the regulation of immune responses. The goal of this study was to evaluate the potential of radiofluorinated carboximidamides as selective PET radioligands for IDO1. Specific binding correlated with IDO1 expression as measured through *in vitro*, microPET experiments. Specific accumulation of the new radiotracer [^18^F]IDO49 was observed in IDO1-expressing tumors and confirmed by Western blot and IHC analyses. These results suggest that [^18^F]IDO49 has substantial potential as an imaging agent that targets IDO1 in tumors, and therefore may be utilized as a companion diagnostic for IDO1 targeted therapies.

## INTRODUCTION

The enzyme indoleamine 2,3-dioxygenase (IDO) regulates immune responses through the capacity to degrade the essential amino acid tryptophan (Trp) into kynurenine (Kyn) and other downstream metabolites. The depletion of Trp and resulting accumulation of Kyn suppresses effector T-cell (T_eff_) function and favors the differentiation of immune inhibitor regulatory T (T_reg_) cells. Current experimental evidence indicates that IDO can be expressed by a variety of cell types, including dendritic cells, tumors cells and stromal cells [1]. The studies have led to the hypothesis that the IDO pathway is a key regulatory element responsible for induction and maintenance of peripheral immune tolerance in normal physiological situations as well as in pathological conditions including autoimmunity, neuropathology, infection and cancer [2]. Recent studies have consistently shown expression of IDO in a variety of resected human extra-cerebral tumors, including lung cancer [3, 4], colorectal cancer [5, 6], breast cancer [7], hepatocellular carcinoma [8], prostate cancer [5], pancreatic carcinoma [5], and ovarian cancer [1]. Several of these studies demonstrated that high expression of IDO was associated with a reduced survival [3, 6, 9]. These findings suggest that IDO could be an important prognostic marker in a variety of tumors, and may be useful as a predictive biomarker for response to selective IDO inhibitors as immunotherapeutic agents.

IDO has two isoforms, IDO1 and the recently discovered IDO2, both are expressed in tumors and tumor-draining lymph nodes [10, 11]. As the discovery of the IDO2 enzyme is so recent, its substrate panel is still yet to be defined. The little activity of IDO2 in the *in vitro* assay also slows down the study of its substrate and inhibitor [10, 12]. Considering IDO1 can recognize a spectrum of indoleamine derivatives (L-tryptophan, D-tryptophan, 5-hydroxy-tryptamine, tryptamine and serotonin), IDO2, an isoform of IDO1, may recognize broad substrates. Therefore, using radiolabeled substrate α-[^11^C] methyl-L-tryptophan may result in the IDO profile which is the merge profiles of IDO1 and IDO2 activities. Using radiolabeled specific enzyme inhibitors with selective binding to the targeted enzyme (e.g. IDO1) may solve the problem and have potential to get *in vivo* enzyme activity profiles of IDO1.

Currently, there are no available response biomarkers for IDO targeted therapies, such as 1-D-MT [13], INCB24360 [14], and NLG919 as IDO1 targeted therapies in clinical trials [13–16]. IDO1 expression can be approximated by measuring the serum concentration ratio of Kyn to Trp (K/T) or via analysis of biopsy samples [17]. However, serum K/T only reflects average IDO1 expression but not localized activities. Moreover, other enzymes such as tryptophan 2, 3-dioxygenase (TDO) and indoleamine 2, 3-dioxygenase-2 (IDO2) also affect Trp and Kyn levels because they catalyze the same reaction. Analysis of biopsy samples using immunohistochemistry (IHC) can quantify IDO1 protein expression and RT-PCR can quantify IDO1 mRNA expression, but these invasive methods cannot be collected longitudinally.

Positron emission tomography (PET) is a powerful molecular imaging tool that allows non-invasive, and longitudinal *in vivo* measurements of multiple molecular processes in various organs using radiolabeled tracers. A PET imaging tracer that is specific for IDO1 would allow noninvasive detection of IDO1 levels, which would have potential applications for variety of cancer detection and staging, and could also provide a new approach for predicting and monitoring the role of IDO1 in immunotherapy. It is envisioned that IDO-PET could identify patients most likely to respond to IDO-targeted therapy. Additionally, IDO-PET could be used to measure and adjust the tumor response during therapy. A non-metabolizable IDO1 substrate (α-methyl Trp, AMT) has been reported to target brain tumors with different profiles of IDO1 expression [18]. Alpha-[^11^C] methyl-L-tryptophan (^11^C-AMT), an IDO1 substrate, has been identified as a good PET tracer for the kynurenine pathway [19]. However, IDO1 is only involved in the first step of the kynurenine pathway. Increased ^11^C-AMT cellular uptake is a complicated process involving many enzymes in both tryptophan transport and metabolism. Further, the short half-life of ^11^C is ill-suited to allow steady-state biodistribution and limits utility to only a few centers. If IDO1-specific inhibitors can be radiolabeled with the more widely available fluorine-18, the new PET probes could measure IDO1 levels *in vivo*. Further, these new molecular probes could establish a practical approach for predicting and monitoring vaccine immunotherapy in the clinic. Here, we report radiofluorinated carboximidamides based on the IDO1 inhibitor, INCB024360, as IDO1 targeted tracers for tumor imaging with PET. Two lead compounds, [^18^F]IDO5L and [^18^F]IDO49, were evaluated by *in vitro* and *in vivo* assays including stability, cell occupancy measurements, western blotting and IDO1 immunohistochemistry of tumors. These were evaluated in induced HeLa tumor bearing mice wherein IDO1 was induced with IFN-γ and showed high correlation with IDO1 expression and [^18^F]IDO49 tracer uptake.

## RESULTS

### Synthesis of carboximidamides analogs

**Scheme 1:**
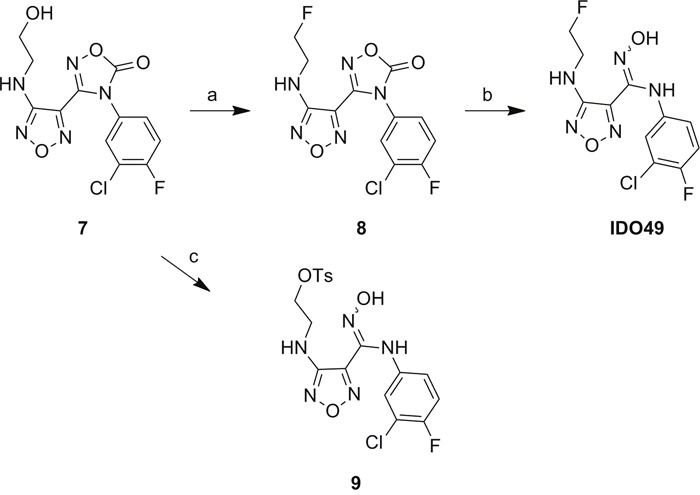
Synthesis of the reference compound IDO49 and precursor 9 a. Methyl DAST, Dichloromethane, 40 °C, overnight, 81%; b. NaOH, Tetrahydrofuran, R.T. 1h, 98%; c. 4-Toluenesulfonyl chloride, Et_3_N, 0°C to R.T., overnight, 66%.

The unlabeled IDO1 inhibitor reference compound, IDO5L, was synthesized based on the structure of 4-Amino-1,2,5-Oxadiazole-3-Carboximidamide as previously reported [20]. The reference compound IDO49 (N-(3-chloro-4-fluorophenyl)-4-((2-fluoroethyl)amino)-N'-hydroxy-1,2,5-oxadiazole-3-carboximidamide) and the tosylate precursor 9 (2-((4-(N-(3-chloro-4-fluorophenyl)-N'-hydroxycarbamimidoyl)-1,2,5-oxadiazol-3-yl)amino)ethyl 4-methylbenzenesulfonate) were synthesized from compound 7 which is illustrated in Scheme 1. The alcohol 7 was fluorinated by Methyl DAST (Dimethylaminosulfur trifluoride) to give compound 8 in 81% yield. Then the oxadiazolone ring was hydrolyzed with sodium hydroxide to yield the amidoxime, IDO49, in 98% yield. The tosylate precursor 9 was synthesized by coupling compound 7 with p-toluenesulfonyl chloride under basic conditions in 66% yield. Compound 7 was synthesized from compound 1 using the reported method with minor modifications shown in Supplementary Scheme 1 [20]. Chloro-oxime 1 was coupled with amine to yield amidoxime 2 which was converted to amidoxime 3 by overnight reflux in aqueous potassium hydroxide. Amidoxime 3 was then activated to chloro-oxime 4 and subsequently coupled with 3-chloro-4-fluoroaniline to provide compound 5 in a 76%, 4-step overall yield. The amidoxime of compound 5 was protected as oxadiazolone 6 using 1,1’-carbonyl diimidazole in 94% yield. Finally, the methoxy group was removed by boron tribromide to yield alcohol 7 in 82% yield. The supplemental data contains detailed experimental procedures.

### *In vitro* characterization

**Figure 1:**
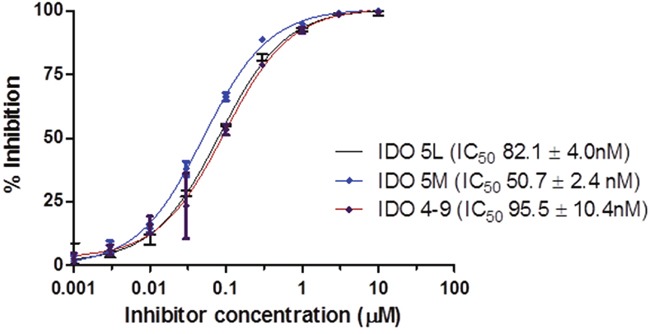
IDO enzyme inhibition assay (Each data point reflects the mean value of n ≥ 3, error bars show standard deviation from the mean).

**Figure 2:**
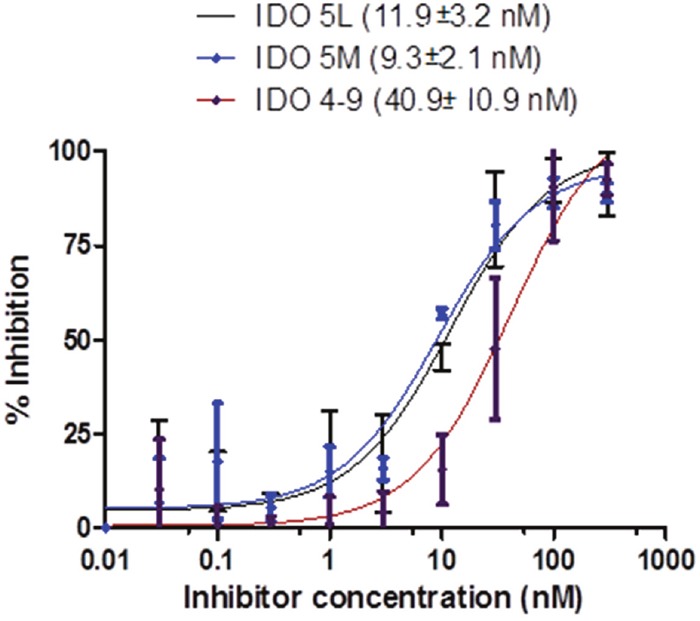
IFN-γ induces IDO activity in the HeLa cell lines, the activity of which is inhibited by different IDO inhibitors (IDO49, IDO5l, IDO5m)

**Figure 3:**
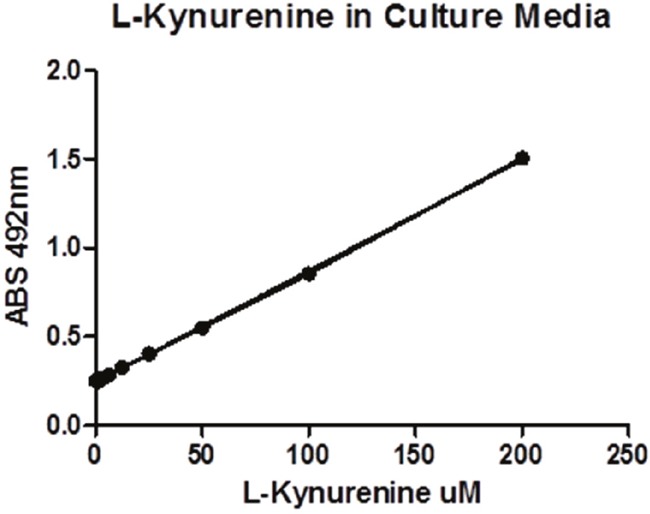
The standard curve of L-Kynurenine's UV absorbance under various concentrations

The binding affinity of the caboxyimidamide analogs to IDO1 was determined through *in vitro* enzymatic assays measuring kynurenine formation in HeLa cells spectrophotometrically. Compounds IDO5L and IDO49 were tested. The data quantify enzyme activity for three different inhibitor concentrations, shown in Figures 1, 2, and 3. These results suggest a higher affinity of human IDO1 for IDO5L and IDO49. IDO5M was shown to be significantly more potent than the IDO49 and IDO5L.

### Radiochemistry

**Figure 4:**
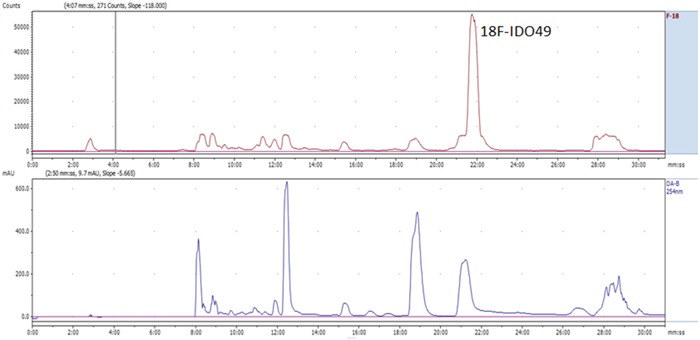
Representative chromatogram from the semi-preparative HPLC separation of the [^18^F]IDO49 product

**Figure 5:**
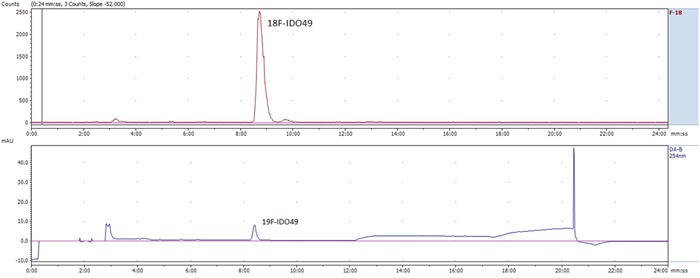
Representative chromatogram from the HPLC analysis of the purified [^18^F]IDO49, co-injection with reference IDO49

Radiochemical synthesis of [^18^F]IDO49 was performed by reacting the tosylate precursor. IDO47 with ^18^F followed by HPLC purification (t_R_ = 20.5-22.8min min) to afford compound [^18^F]IDO49 (Figure 4). The radioligand was isolated in a decay corrected radiochemical yield of 62.8 ± 3.1% (n=5), radiochemical purity of 97.5%, and specific activity (1.1 Ci/umol) of the product. [^18^F]IDO49 ws characterized by analytical HPLC with the retention time of 8.8 min. The identity of [^18^F]IDO49 was confirmed by a co-injection with an nonradioactive standard IDO49 (Figure 5).

### Lipophilicity

Lipophilicity of [^18^F]IDO49 was measured using the shake-flask method (n-octanol/buffer pH 7.4 partition coefficient) and a calculated LogP (cLogP, n-octanol/water partition coefficient) was determined using CHEMBIODRAW software. The experiments determined a logD_7.4_for [^18^F]IDO49 of 1.37 ± 0.02 (n=3) and clogP was 2.79. Using the same method, the a logD_7.4_ of [^18^F]IDO5L was 2.13 ± 0.11 (n=3).

### *In vitro* stability

**Figure 6:**
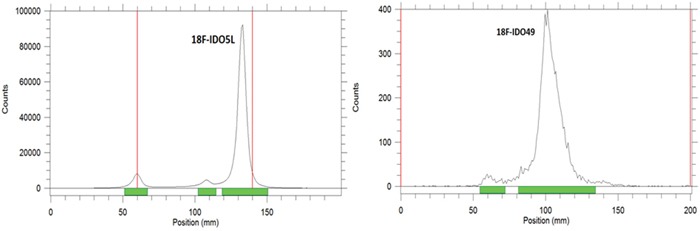
Radio-TLC profile of [^18^F]IDO5L and [^18^F]IDO49 after incubation in physiological saline at 37°C for 3 h

The *in vitro* stabilities of [^18^F]IDO5L and [^18^F]IDO49 were evaluated by radio-TLC. As shown in Figure 6, after incubation in mouse serum at 37°C for 3 h, >95% of the radioactivity was observed in the intact radioligands, [^18^F]IDO5L and [^18^F]IDO49. This analysis confirmed the absence of radioactive degradation products after 3 h in mouse serum at 37°C.

### Hela cell uptake of radioligands and competitive cell-binding assay

**Figure 7:**
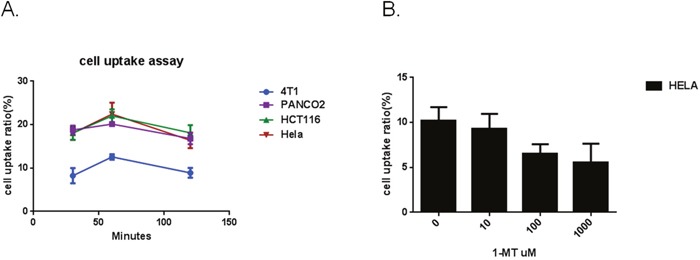
**A**. Cellular uptake of [^18^F]IDO5L at 30 mins, 60 mins and 120 mins. **B**. Cellular uptake of [^18^F]IDO5L by Hela at 120 mins with the inhibitor of 1-L-MT.

**Figure 8:**
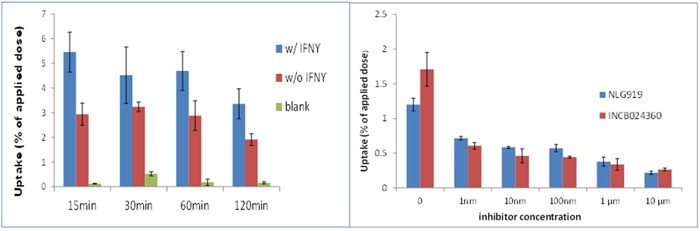
Hela Cell uptake of [^18^F]IDO49 assays ( all cells treated by IFN with inhibitor) INCB024360 (IC50 = 10 nM) NLG919

To investigate IFNγ-induced IDO1 expression in multiple cell lines, enzyme expression was measured in HeLa, PANC02, HCT116 and 4T1 cells. Those cells were cultured in the absence or presence of IFNγ, followed by Western Blotting to confirm IDO1 expression. [^18^F]IDO5L uptake in Hela, PANC02, HCT116, and 4T1 cells was assayed at 30, 60, and 120 mins of incubation (n=3) (Figure 7). Cellular uptake peaked at 60 mins for all cell lines. Time-dependent uptake of [^18^F]IDO5L was compared directly with IFN-γ stimulated HeLa, PANC02, HCT116 and 4T1 cells. Uptake of [^18^F]IDO5L was greater than specific activity uptake in HeLa cells, PANC02 and HCT116 in all time points, and matched the same inhibitor Hela cell assay in the previous report [21]; Since IDO1 expression is selectively inhibited by 1-L-MT in HeLa cells [21], we tested the uptake of [^18^F]IDO5L by HeLa at 120 min in the presence of 1-L-MT (n=4), the results showed there was strong inhibition of IDO activity with high level of 1000 μM 1-L-MT suggesting that our [^18^F]IDO5L is a much better inhibitor for uptake via the IDO1 expression Hela cell line. We also evaluated uptake of [^18^F]IDO49 in IFN-γ stimulated HeLa cells with different time points, the results show [^18^F]IDO49 was retained at favorable levels in IDO1 expression Hela cell line with IFN-γ stimulation. We performed a further competitive cell-binding assay on Hela cells, using IDO1 inhibitors NLG919 and INCB024360 as competitive ligands. When co-incubated with both IDO1 inhibitors, [^18^F]IDO49 uptake in Hela cells decreased in a dose-dependent manner (Figure 8). The cell uptake and competitive cell-binding assay results elucidated the binding of [^18^F]IDO49 to IDO1.

We confirmed the specific uptake of [^18^F]IDO49 in IDO1 expressed IFN-γ stimulated HeLa cells. Specific binding also was determined by blocking with INCB024360 and an alternative competitive IDO1 inhibitor, NLG919. We found [^18^F]IDO49 was capable of binding IDO1 with high specific binding *in vitro* (Figure 8).

### Small animal PET imaging

**Figure 9:**
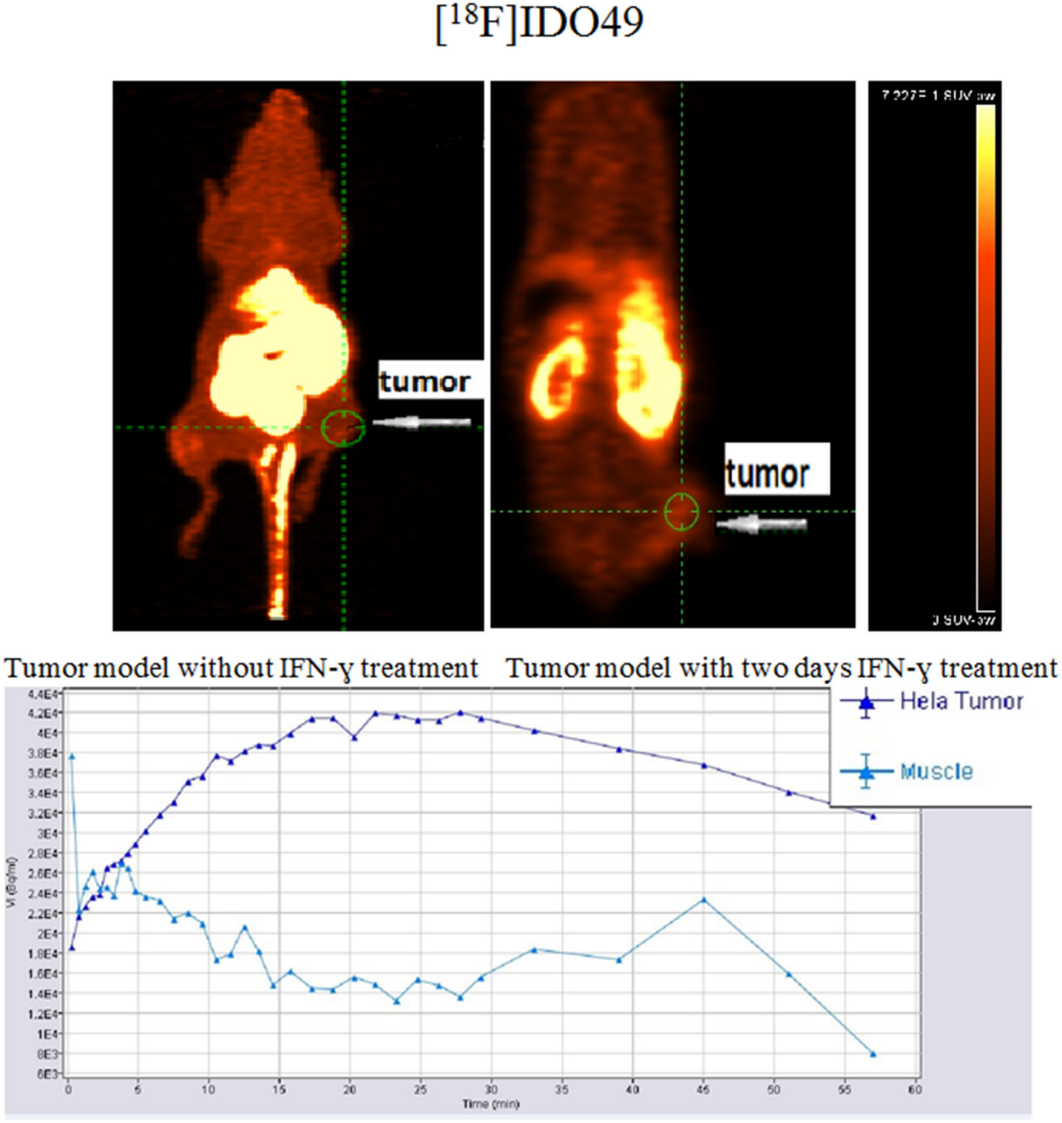
[^18^F]IDO49 PET imaging in HeLa Cervical tumor model with IFN-γ treatment

**Figure 10:**
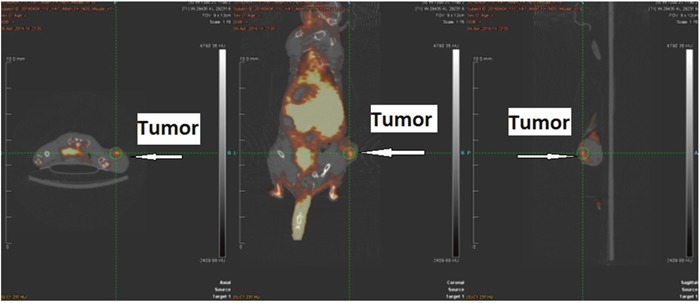
[^11^C]AMT PET imaging in HeLa Cervical tumor model with IFN-γ treatment

To determine the feasibility of [^18^F]IDO49 for noninvasive detection of rHu IFN- γ treated tumors, whole body PET images for [^18^F]IDO49 were obtained with and without rHu IFN- γ treatment in mice bearing HeLa xenografts. Representative late summed images from 60 min dynamic microPET imaging studies of IFN- γ treated and untreated tumor bearing mice administered [^18^F]IDO49 were shown in Figure 9. For the IFN- γ treated tumor bearing mice, the tumor radioactivity uptake of [^18^F]IDO49 was visualized 60 min postinjection. Radioactive uptake was high in the kidney and other radioactivity accumulation was observed in liver. Tumor bearing mice treated with [^18^F]IDO49 but without IFN- γ were also imaged by PET with a 60 min dynamic scan (Figure 9). These mice had lower activity in the same tumor region compared with the 3 days IFN- γ treated mice. SUV analysis of the summed images from the dynamic microPET scans confirmed the visual assessment of the images. The average SUV of the tumor with IFN- γ treatment mouse for [^18^F]IDO49 was higher than untreated tumor mouse. The tumor to muscle (T/M) and relative uptake ratios of [^18^F]IDO49 reached the peak (2.29 ± 0.05, p < 0.05) at 60 min postinjection. While more IDO1 specific binding was seen diffusely when [^11^C]AMT imaging was performed, PET images with [^11^C]AMT of IFN- γ treatment tumor mouse shown in Figure 10, there was significant tracer accumulation were observed in tumor region when compared with same tumor model imaged with [^11^C]AMT.

### Western blotting and IDO IHC of tumors

**Figure 11:**
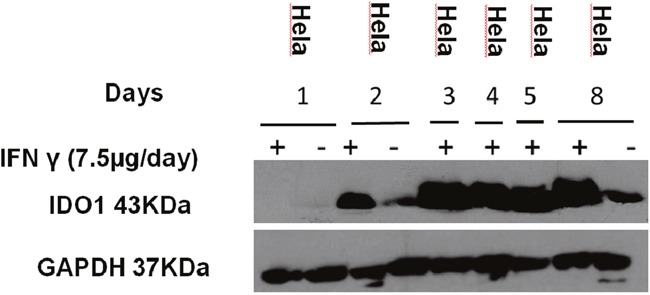
Expression IDO1 of the mice Mice injected with IFN-γ (7.5 μg/day on days 1, 2, 3, 4, 5, 8) and tumor tissues were collected. Protein was extracted from the tumor for the western blotting analysis. (“+” represent inject IFN- γ, “-” represent non-inject IFN-γ).

**Figure 12:**
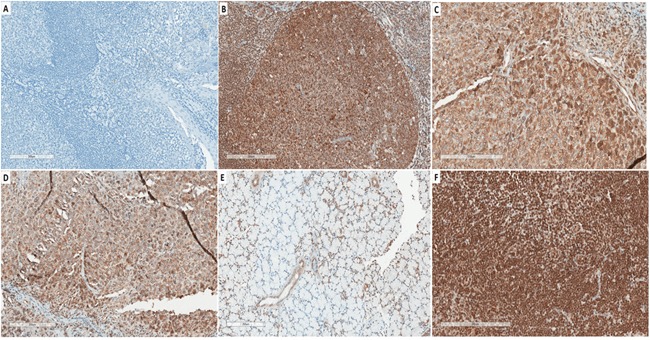
Representative immunohistochemical expression (IHC) of IDO **A**. Tonsil as negative control in saline treated WT. **B**. Tonsil were immunohistochemically stained for the IDO protein as positive control; **C**. at 3 days of IFN-γ -treatment in Hela tumor tissue; **D**. at 8 days of IFN-γ-treatment in Hela tumor tissue. **E**. Thymus from tumor mouse. **F**. Lymph nodes from tumor mouse.

*In vivo* IDO1 expression was evaluated by Western blot. Although there was variation, all tumors with IFN-γ treatment expressed IDO1 protein except day-1 IFN-γ treatment group (Figure 11). The histological features of HeLa tumors were also assessed through IHC and IDO1 staining (Figure 12). In the IFN-γ treated mice, the expression levels of IDO1 were significantly higher in HCCs as compared to the surrounding non-cancerous tissue. IDO1 protein expression was shown to increase for the group of mice treated with IFN-γ over multiple days when compared to the group of mice treated with IFN-γ in single day. Animals without IFN-γ treatment had little expression. These findings, together with those of Western blot suggest that induction of IDO1 might be associated with IFN-γ-treatment in HeLa cancer lesions.

## DISCUSSION

The current practice in anti-tumor immune therapy is focused on the regulation by cell-cell communication of checkpoints and checkpoint ligands, such as PD-1 and PD-L1. In fact, tumor-immune interactions are complex and also involve a significant number of soluble factor inhibitors, such as kyneuranines, which are produced by IDO1. Although others are working in this area [22, 23], this work is based on the premise that imaging of IDO1 expression *in vivo* will inform better decisions during the course of therapy. An enabling aspect of molecular imaging is the ability to capture data longitudinally, i.e. during the course of therapy. As IDO1 is induced by IFN-γ that is released by activated immune cells, it is likely that the efficacy of IDO1 inhibition will be critically dependent on timing. We propose that an IDO1 imaging agent would be enabling and paradigm shifting, as it could be used during the course of therapy to identify the optimum dosing schedule in individual patients. This study investigated the distribution of radiofluorinated carboximidamides as novel imaging tools in tumor-bearing animals with the aim to determine specific accumulation in IDO1 expressing tumors. We assessed the uptake of these tracers *in vitro* in IDO1 expressing tumor cell lines and also evaluated [^18^F]IDO49 in IDO1-expressing tumor models using microPET.

Among the newly developed IDO1 inhibitors reported so far [21], INCB024360 is an orally bioavailable small molecule inhibitor of IDO1 that has nanomolar potency (IC50 =7.1 nM) in both biochemical and cellular assays, potent activity in enhancing T lymphocyte, dendritic cell and natural killer cell responses *in vitro*, with a high degree of selectivity. The Phase I dose-escalation trial demonstrated that INCB024360 results in greater than 90 percent inhibition of IDO1 activity at generally well-tolerated doses [24–26]. Moreover, the rapid clearance rate (t_1/2_< 0.5h) of INCB024360 leads to excellent tumor-to-normal tissue contrast, which is preferred for a PET imaging agent. Radio-fluorination of carboximidamides, such as INCB024360 analogs, could make them ideal targets for IDO1-specific PET imaging. In the present study, we developed novel targeted PET imaging probes for the *in vivo* detection of IDO1 protein in a cervical cancer mouse model.

In order to achieve these objectives, the carboximidamides compounds were synthesized as INCB024360 analogs, including reference compounds IDO5m, IDO5L and IDO49. Radiolabeling precursors of both IDO5L and IDO49 also were synthesized using two different approaches both easily transposable for the radiosynthesis. We investigated the potential influence of IDO5L and IDO49 on the binding affinity to IDO1 *in vitro*. We did observe that both compounds are slightly more potent in the more physiologically relevant HeLa cell-based assays than in the enzyme assays. This may be due to the complexity of the enzyme assay which utilizes a methylene blue ascorbate regeneration system to maintain IDO1 in the active reduced form or unidentified differences between the recombinant IDO1 used in the enzyme assay and native IDO1 in HeLa cells. Nevertheless, a good correlation was established between the two assays. The results establish IDO5L as the most potent IDO1 inhibitor of the series, and its IC_50_ was determined as 11.9 nM compared with the previously reported IC50 of 19.0 nM [21]. IDO49 displayed high affinity for IDO1 with a mean IC50 of 11.9 nM, which is only slightly weaker than the affinity of INCB24360 (7.1 nM), suggesting that attachment of the fluoroethyl moiety has only a minor effect on IDO1 binding affinity, kinetic analysis demonstrated that three carboximidamides analogs are potent inhibitors of IDO1.

Radiochemical synthesis of [^18^F]IDO5l was performed in multiple steps in our previous report [20], in this study, [^18^F]IDO49 was designed with a single step radiolabeling procedure which is easily separated and has high radiochemical yield. It should be emphasized that, to our knowledge, the radiofluorinated carboximidamide analogs described here are the first IDO1-targeted PET agents to be developed based on the inhibitor. Indeed, a variety of tryptophan based ^11^C- and ^18^F-labelled PET agents have already been shown to produce highly defined images of the kynurenine pathway in animal tumor models and human studies [27–30]. Kramer etc. investigated how ^18^F-L-FEHTP accumulates in endocrine and nonendocrine tumor models

via LAT1 transport but is not decarboxylated by AADC; ^18^F-L-FEHTP may serve as a PET probe for tumor imaging and quantification of tumor LAT1 activity [31]. Henrottin etc. evaluated 1-[^18^F]FE-DL-Trp using *in vitro* enzymatic assays, *in vitro* studies and showed that L-[^18^F]5 may be a good substrate of hIDO [30]. Other groups tested kynurenine pathway of tryptophan metabolism using 1-L-[^18^F]FETrp *in vivo* [32]. Among these substrates of IDO1, LAT1 or TDO, specific binding affinity for IDO1 proteins in animals or humans is still not clear. [^11^C]AMT can be metabolized by IDO1 or TDO via the kynurenine pathway. Tumoral accumulation of [^11^C]AMT tracers can occur as a result of tumoral transport, in which high LAT1(L-type amino acid transporter 1) expression may play a central role. Our motivation for seeking yet another inhibitor based IDO1-targeted PET agent was prompted by the need to create an imaging agent that could meet the economic requirements for translation of PET agents into the clinic. Fluorine-18 was selected ahead of ^11^C not only because of the higher resolution images that it yields but also because of the extensive production and distribution networks that already exists for fluorine-18.

Because it is attractive as a one-step radiolabeling procedure, tosylate precursors are commonly employed for a nucleophilic substitution of no-carrier-added [^18^F] fluoride [33, 34]. This type of precursor can be used for an ^18^F-labeling strategy to replace a conventional complex and long process of multiple-step radiolabeling procedure, reducing reaction time and labor significantly. We prepared a new ^18^F-labeled, IDO1-targeted imaging agent, [^18^F]IDO49, from the tosylate precursor IDO47. The one-pot, two-step radiosynthesis of [^18^F]IDO49 was performed under mild reaction conditions and in radiochemical yields suitable for clinical studies. Furthermore, unlike the radiosyntheses of [^18^F]IDO5L, no intermediate purification or coupling steps were required, since a sample hydrolysis step in radiolabeling procedure, a semipreparative purification by HPLC was needed in final step only. The radiochemical yield and reaction times were also optimized for these minimal reactions (data not show, making them transferable to automated synthesis.

High affinity for the imaging target is essential for achieving a specific signal. Agent lipophilicity is another important variable with implications in tumor perfusion, biodistribution, and pharmacokinetics. Compounds with high lipophilicity can be hampered by slow clearance, non-specific binding, and lipophilic radiometabolites. With these factors in mind, we selected [^18^F]IDO49 ligands based on their affinity, radiochemical yield, ease of synthesis, and lipophilicity for the first imaging study.

PET images of HeLa tumor bearing mice showed [^18^F]IDO49 selectively accumulated in tumors with IFN- γ treatment. Indeed, [^18^F]IDO49 PET imaging confirmed IFN- γ treatment significantly induced IDO1 expression in HeLa tumors. IFN-γ is released by activated immune cells which stimulates more IDO1 expression, and this hypothesis was confirmed by our studies of histopathology and IDO1 IHC of tumors. Since IDO1 activity has been established as one mechanism by which tumors protect themselves against the host's immune response [35], we assessed mouse models with IFN-γ induction. Our *in vivo* studies demonstrate that IDO1 activity depends on IFN-γ stimulation and is regulated in a tissue-specific manner. IFN-γ may play a critical role in activating macrophages, lymphocytes other cells. Thus, our use of HeLa tumor bearing models with IFN- γ treatment enabled us to confirm that [^18^F]IDO49 accumulation in the IFN-γ treatment tumor mouse was due to *in vivo* targeting of activated IDO1 expression rather than nonspecific retention. Some *in vitro* studies indicate that IFN-γ induced IDO1 activities are involved in IDO dependent mechanisms, which is mainly induced by IFN-γ. IDO1 converts L- tryptophan to N-formylkynurenine, and strong IDO1 induction results in L- tryptophan depletion [4, 36, 37]. Our [^11^C]-AMT PET imaging confirmed that IFN- γ treatment of HeLa cells primarily express the IDO1 to convert tryptophan via the kynurenine pathway. Further studies will be necessary to assess expression of TDO and LAT1 to clarify the correlation of the kynurenine pathway. From this initial PET imaging study, there were not significantly uptake of [^11^C]-AMT and [^18^F]IDO49 in lymph nodes of HeLa tumor bearing models, which display positive IDO1 expression in IHC, it may need a longer PET acquisition time to obtain an accurate imaging in lymph nodes. To our knowledge, this study is the first time an IFN-γ treated, murine tumor model has been used to identify IDO1 specificity with a PET imaging probe. We imaged the tumors with the carboximidamide analog radioligand, [^18^F]IDO49. This radioligand assessed the distribution and intensity of IDO1 expression. Future studies will explore the radioligand in new tumor models and encourage us to explore the translation of [^18^F]IDO49 into clinical trials.

In this study, we synthesized novel radiofluorinated carboximidamides as selective IDO1 enzyme radioligands. The specific binding of these compounds correlated with *in vitro* IDO1 expression. MicroPET experiments indicated the radioligand [^18^F]IDO49 specifically accumulated in IDO1-expressing tumors which were orthogonally confirmed by Western blot and IHC analysis. A murine HeLa tumor model with IFN- γ treatment, enabled us to confirm the [^18^F]IDO49 accumulation in IDO1 positive tumors. These results have implications that [^18^F]IDO49 has substantial potential as an imaging agent targeting IDO1 in tumors.

## MATERIALS AND METHODS

### Organic synthesis

The precursors and reference compounds IDO5L and IDO5M were synthesized as previously described [20]. The synthesis of the corresponding reference compound IDO49 and tosylated precursor is described in the supplemental information. All other chemicals and materials were obtained from commercial sources, were of analytic grade, and were used as received.

### IDO enzyme assay

Human IDO (enzolifesciences.com ALX-201-333-C050) performed the oxidative cleavage of the pyrrole ring of the indole nucleus of tryptophan to yield *N*’-formylkynurenine. The assays were performed at room temperature as described as literature reported with minor revision [38]. Briefly, in each well of a 96 well-plate, 10 μL of human IDO (0.05mg/mL in KHPO_4_, 50mM, PH 6.5) was added into 39 μL buffer (KHPO_4_, 50mM, PH 6.5) and 1 μL inhibitor buffer in DMSO (2000, 600, 200, 60, 20, 6, 2, 0.6, 0.2 μM). Then, 50 μL of substrate buffer (4mM L-tryptophan (#T0254, Sigma), 40mM ascorbate, 20uM methylene blue (#M44907, Sigma), 0.2mg/mL catalase (#C30, Sigma)) was mixed into each well and incubated at 37 °C for 2 hours. Then 10 μL of 6.1 N trichloroacetic acid (#T0699, Sigma) was mixed into each well and incubated at 52 °C for 30 min to hydrolyze *N-*formylkynurenine and produce kynurenine. The reaction mixture was incubated with 100 μL of 0.02 g/mL *p*-(dimethylamino)benzaldehyde (#mk1836100, Fisher) in acetic acid at room temperature for 10 minutes. The yellow color derived from kynurenine was measured at 492 nm using a microplate reader. L-Kynurenine (#K8625, Sigma) used as the standard and prepared in a series of concentrations (1000, 500, 200, 100, 50, 20, 10 μM). The percent inhibition at individual concentrations was determined and the average values of duplicates were obtained. The data was processed using nonlinear regression to generate IC50 values (Prism Graphpad).

### IDO Hela cells assay

The Hela cell assay was carried out according to the reference [21, 38] with minor modification. HeLa cells were obtained from *Springett Lab, Moffitt Cancer Center* and routinely maintained in Earle's Minimum Essential Medium (EMEM) (ATCC 30-2003) and 10 % fetal bovine serum. Cells were kept at 37 °C in a humidified incubator supplied with 5% CO_2_. The assay was performed as follows: HeLa cells were seeded in a 96 well culture plate at a density of 5 × 10^3^ per well in 100 μL culture media. After overnight incubation (16 h), human IFN-γ (50 ng/mL, final concentration) and serial dilutions of compounds in 100 μL culture medium per well were added to the cells. After an additional 48 h incubation, 140 μL of the supernatant per well was transferred to a new 96 well plate. Ten microliters of 6.1 N trichloroacetic acid (#T0699, Sigma) were mixed into each well and incubated at 50 °C for 30 min to hydrolyze *N-*formylkynurenine to produce kynurenine. The reaction mixture was then centrifuged for 10 min at 2500 rpm to remove sediments. One hundred microliters of each supernatant were transferred to another 96 well plate and mixed with 100 μL of 2% (w/v) *p*-dimethylaminobenzaldehyde (#15647-7, Sigma-Aldrich) in acetic acid. The yellow color derived from kynurenine was measured at 492 nm using a SPECTRAmax 250 microplate reader. L-Kynurenine (#K8625, Sigma), used as the standard, was prepared in a series of concentrations (200, 100, 50, 24, 12.5, 6.3, 3.2, 1.6 μM) in 100 μL HeLa cell culture media and analyzed in the same procedure. The percent inhibition at individual concentrations was determined and the average values of duplicates were obtained. The data was processed using nonlinear regression to generate IC_50_ values (Prism Graphpad).

### Radioligand preparation

**Scheme 2:**
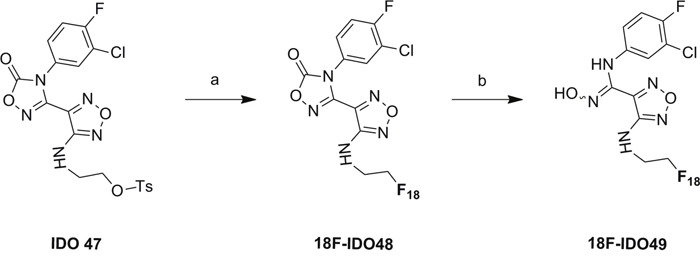
Radiosynthesis of ^18^F-IDO49 Conditions: a. [^18^F]KF/K222, MeCN, RT, 90 °C, 5 min. b. NaOH(aq.), 15 min, R.T.

[^18^F]IDO5L [20] and [^11^C]AMT [27] were synthesized as previously described. The synthesis of the target tracer [^18^F]IDO49 (Scheme 2) was performed by the conventional Kryptofix-mediated nucleophilic ^18^F-substitution of tosylated precursor IDO47 followed by NaOH hydrolysis, and the labeling yield was determined by analytical HPLC. Aqueous [^18^F]fluoride (40-60 mCi) was trapped on a pre-conditioning QMA cartridge and eluted with a mixture of Kryptofix [2.2.2] (800 μL of a 22.6 mg/mL stock solution in MeCN) and K_2_CO_3_ stock solution (50 μL of a 84 mg/mL stock solution in water). [^18^F]Fluoride was dried at 120 °C under a stream of nitrogen by azeotropic drying with anhydrous acetonitrile (3 × 0.3 mL) to give the no-carrier-added [K/K222] + ^18^F- complex as a white semi-solid residue. After cooling to room temperature, a solution of tosylate precursor IDO47 (5.0 mg, 20 μmol) in anhydrous DMSO (0.5 mL) was added into the reaction vial. After heating at 90 °C for 5 min, the mixture turned a yellow color. After cooling to the room temperature, aqueous NaOH (2 N 0.1 mL) was added to the reaction mixture to hydrolyze the product. The reaction mixture stirred at room temperature for 15 min and water (8.0 mL) was added. The aqueous solution was then passed through an activated C18 Sep-Pak plus (Waters Corp). The Sep-Pak was rinsed with water (8.0 mL) and the residue solvent was partly removed by air (20 mL). The product ([^18^F]IDO49) was slowly eluted through the column with acetonitrile (1.5 mL). After solvent reduced to ~ 0.2 mL by a stream of nitrogen in 100 °C, water was added (0.7 mL) and the mixture was purified by semi-preparative HPLC. The collected HPLC fraction (~4 mL) with a retention time of 20.5-22.8 min was diluted with water and then passed through the activated C18 Sep-Pak column. After washing with 4 mL of water, the labeled product was eluted by 1.5 mL acetonitrile and dried under a stream of nitrogen in 100 °C. Finally, the residue was dissolved in 0.3 mL physiological saline for the animal experiments.

### Determination of radiochemical purity and specific radioactivity

Chemical and radiochemical purities were assessed by HPLC analysis. Radioligand product was chromatographed on the HPLC under linear gradient conditions. Concentration was determined from the integration of the UV absorbance corresponding to the radiolabeled product and the application of a linear regression from a standard curve of reference compound. The experiments were completed in triplicate. Specific activity of the radioligand was calculated from the ratio of measured radioligand concentration and activity injected on the HPLC (determined from wipe counter). HPLC analysis was performed using an Agilent 1260 system with in-line UV detector (254 nm) and a NaI crystal flow-count radioactivity detector (Lablogic Flow-RAM detector). The analytical column was an Agilent Eclipse XDB C18 column (5 μm, 4.6 × 250 mm) with the flow rate 1.0 mL/min using MeCN/0.1% acetic acid in H2O 50/50, 12/88, or 40/60 as an eluent. Retention time was 11.5 minutes for [^18^F]IDO49.

### Lipophilicity determination

[^18^F]IDO49 (2-30 kBq in 30μL of water) was added to a two-layer system of n-octanol (500 μL) and 0.1 M phosphate buffered saline (PBS) pH 7.4 (500 μL) in an Eppendorf vial. The vessel was vortexed for 3min and then centrifuged at 10,000 rpm for 2 min. An aliquot of each layer (100 μL) was assessed for radioactivity in a wipe counter. The partition coefficient (logD_7.4_) was calculated as the decimal logarithm of the ratio between the counted radioactivity in the n-octanol layer and the counted radioactivity in the aqueous layer (count of n-octanol sample – count of background) / (count of PBS sample – count of background). CLogP (calculated LogP) values were determined using CHEMBIODRAW ULTRA 12.0 software (Cambridge soft. Perkin-Elmer, Waltham, MA, USA).

### *In vitro* stability

To examine *in vitro* stability, approximately 400 μL of [^18^F]IDO5L or [^18^F]IDO49 was mixed with 2 mL of mouse serum at 37°C and incubated for 3 h. Two-hundred microliter samples were collected at 3 h and analyzed by radio-TLC.

### Hela cell uptake of radioligands

HeLa cells were seeded in a 24 well culture plate at a density of 2 × 10^4^ per well in 400 μL culture media w/ FBS (n=3). After overnight incubation (16 h), human IFN-γ (25ng/well, 50 ng/mL final concentration) in 100 μL DMEM with FBS were added into cells. The cells were rinsed with phosphate-buffered saline (PBS), 500 μL of Earle's Minimum Essential Medium (EMEM) (ATCC 30-2003), and 10 % fetal bovine serum was added to the culture wells. [^18^F]IDO5L and [^18^F]IDO49 (2 μCi/well, n=3) were then added to the wells and incubated for 4 time points (15, 30, 60, and 120 min) in triplicate. At each given time point, the cell supernatants were discarded and cells were washed 3 times with PBS (0.5 mL). Next the cells were lysed with 150 μL of trypsin-EDTA in each well and incubated for 5 min at 37 °C. Then the cell digestion solution was collected and mixed with another 150 μL of PBS into each well. All measurements were performed with a γ-counter (Wizard; PerkinElmer).

### Competitive cell-binding assay

The binding affinities and specificities of [^18^F]IDO5L and [^18^F]IDO49 were determined using IDO1 inhibitors NLG919 and INCB024360 as competitive ligands, HeLa cell preparation procedures were the same as above. To each well were added increasing concentrations of NLG919 and INCB024360 (1-10000 nM) in fresh medium (0.5 mL). After incubating for 15 min at 37 °C, [^18^F]IDO5L and [^18^F]IDO49 (2 μCi/well, n=3) were added to the wells, and incubated for 30 minutes separately. Then the cells were rinsed with PBS (3 × 0.5 mL) to remove any unbound radioactive materials, subsequently lysed with 150 μL of trypsin-EDTA, and incubated for 5 min at 37 °C. Finally the cell digestion solution was collected and mixed with another 150 μL of PBS into each well. All measurements were performed with a γ-counter (Wizard; PerkinElmer). The specificities of radioligands were determined as a percentage.

### Tumor implantation in mice

All procedures involving animals were reviewed and approved by the Institutional Animal Care and Use Committee (IACUC) at Moffitt Cancer Center. HeLa cells were routinely maintained in Earle's Minimum Essential Medium (EMEM) (ATCC 30-2003) and 10 % fetal bovine serum. Cells were kept at 37 °C in a humidified incubator supplied with 5% CO2. The growth media was changed 2 or 3 times per week and the cells were subcultured at a ratio of 1:10 when needed.

Female athymic C57BL/6 nude mice (4-6 weeks old) were ordered from Charles River Laboratories (Stone Ridge, NY, U.S.A.) and housed in a temperature and humidity controlled room. After 7 to 14 days of acclimatization, a total of 5 ×10^6^ Hela cells were inoculated subcutaneously in 200 μL serum-free medium into the right flank of hind leg. Once tumors reached an appropriate size (3-4 mm in diameter) the mice were randomized into 5 groups (4/group). Then the mice in each group were randomized into two subgroups (rHu IFN- γ treatment or no rHu IFN- γ treatment). All the rHu IFN- γ treatment mice were injected i.p. of 1.5×10^5^ U rHu IFN- γ /mouse/day. Group one mice were anesthetized by gas inhalation (2% vol/vol isoflurane in oxygen) 24 h later. Then the mice were euthanized and tumor tissues were dissected for further IDO1 western blotting and IHC. The same operation was repeated at 48h, 72h, 96h and 120h points.

### MicroPET imaging

In order to evaluate the *in vivo* imaging performance of [^18^F]IDO49 to IDO1 tumors, microPET imaging was performed on nude mice bearing Hela tumor xenografts with and without rHu IFN- γ treatment treatment using the Inveon small-animal PET/CT scanner (Siemens). Anesthesia was induced with 4− 5% isoflurane and maintained with 1− 3% isofluorane delivered with a mixed air/oxygen through a closed nose cone throughout the PET imaging session. Mice bearing Hela tumor xenografts with rHu IFN- γ treatment were injected with 3.70-7.40 MBq (100-200 μCi) of [^18^F]IDO49 in 200 μL of saline, and the baseline control mice without rHu IFN- γ treatment were injected with the same radiotracer. PET data were collected for 90 min following radioligand administration and reconstructed into 38 dynamic frames of increasing length (6 X10, 6×20, 4×30, 9×60, 2×180, 8×300, and 3×600 s). For a comparative study, [^11^C]AMT PET imaging scan was also performed on Hela tumor mice at 60 min after intravenous injection of [^11^C]AMT PET (3.7 MBq). The images were reconstructed and the regions of interest (ROIs) were drawn over the tumor and muscle, PET images were converted to percent injected dose per gram (%ID/g of tissue) for evaluation of tumor specificity. Image analysis was performed using the Inevon Research Workplace software.

### Western blotting and IDO immunohistochemistry of tumors

*Western blotting*: Tumors were homogenized in radioimmunoprecipitation assay lysate buffer (consisting of 50 mM Tris-HCl, pH 7.4, 0.1 mM EDTA, 0.1% SDS, 0.15 M NaCl, 1% sodium deoxycholate, and protease inhibitors). After centrifugation at 12,000 × g for 20 min (at 4°C), supernatants were collected. Protein concentrations were determined using a commercial kit based on the Bradford assay. Bovine serum albumin was used as standard. SDS-polyacrylamide gel electrophoresis (one-dimensional) was performed for separation of the proteins, and the gels were subsequently transferred onto Blot PVDF membranes. The PVDF membranes were blocked in a buffered saline solution (0.05 M Tris-HCl and 0.2 M NaCl, pH 7.4) containing 0.1% (v/v) Tween (TBS with 0.5% bovine serum albumin) and 5% non-fat milk (w/v) for 1 hr at room temperature, and then incubated with the primary antibody [anti-IDO diluted at 1:1000; anti-GAPDH antibody diluted at 1:3000; all the primary antibody in TBST containing 1% non-fat milk] for 1 hr at room temperature. The membranes were subsequently rinsed three times (10 min each) with TBST, incubated with HRP-conjugated anti-mouse IgG or HRP-conjugated anti-rabbit IgG (all the second antibody diluted at 1:10000 and all the primary antibody in TBST containing 1% non-fat milk) respectively, for 1 hr at room temperature, then rinsed three times with TBST (10 min each). Secondary antibodies on the membranes were detected with an ECL detection system.

*Immunohistochemical staining*: Tumors were dissected and fixed in 10% (w/v) neutral buffered formalin for 24 hr. Formalin-fixed tissues were processed into paraffin and cut into 5-mm sections on plain slides. Slides were dried for 45 min at 45°C, dewaxed in xylene, and rehydrated through ethanol- graded solutions to water. Slides were stained using a Ventana Discovery XT automated system (Ventana Medical Systems, Tucson, AZ) as per manufacturer's protocol with proprietary reagents. Briefly, slides were deparaffinized on the automated system with EZ Prep solution (Ventana). Heat-induced antigen retrieval method was used in Ribo CC (Ventana). The rabbit primary antibody that reacts to IDO, (ab106134, Abcam, Cambridge, MA) was used at a 1:25 concentration in Dako antibody diluent (Carpenteria, CA) and incubated for 3 hr. The Ventana OmniMap Anti-Rabbit Secondary Antibody was used for 16 min. The detection system used was the Ventana ChromoMap kit and slides were then counterstained with Hematoxylin. Slides were then dehydrated and coverslipped as per normal laboratory protocol. The immunohistochemical images were taken using an Olympus light microscope equipped with a CCD camera (DP70, Olympus).

### Statistical analysis

Data are expressed as the mean ± standard deviation. The means were compared using Student's *t*-test (SPSS 17.0, SPSS, USA). Differences were considered statistically significant at *P* < 0.05.

## SUPPLEMENTARY MATERIALS


