# Supplementary material for: Evaluation of radiofluorinated carboximidamides as potential IDO-targeted PET tracers for cancer imaging
Source: Oncotarget. 2017 Jan 30;8(29):46900–14. doi: 10.18632/oncotarget.14898 (PMC5564531; doi:10.18632/oncotarget.14898)
Supplement: Supplementary file 1 [file oncotarget-08-46900-s001.pdf]

Evaluation of radiofluorinated carboximidamides as potential IDO-targeted PET tracers for cancer imaging

SUPPLEMENTARY DATA

SUPPLEMENTARY MATERIALS

General

All reagents and solvents were purchased from sigma-Aldrich or Fluka and used as received without further purification. Solid-phase extraction cartridges (Sep-Pak QMA, Sep-Pak C18) were purchased from Water Corp., Milford, MA, USA. Column chromatography was performed on silica gel (60 Å, 230-400 mesh, for flash chromatography). Thin layer chromatography was performed on aluminum plates precoated with silica (200 µm, 60 F_{254}), which were visualized either by quenching of ultraviolet fluorescence (λ_{max} = 254 nm) or by iodine stain. ^{1}H and ^{13}C spectra were obtained on a Varian Mercury 400 MHz spectrometer with $CDCl_3$, MeCN-d_3 and DMSO-d_6 as the solvent. All coupling constants were measured in hertz (Hz) and the chemical shifts (δ_H and δ_C) were quoted in parts per million (ppm) relative to the internal standard TMS (δ 0). High resolution mass spectroscopy (HRMS) were carried out on an Agilent 6210 LC–MS (ESI-TOF).

HPLC analysis and purification were performed on Agilent 1260 using an in-line UV detector (254 nm) and a NaI crystal flowcount radioactivity detector (Lablogic FlowRAM detector). The analytical HPLC was performed on an Agilent Eclipse XDB C18 column (5 µm, 4.6 × 250 mm) with the flow rate 1.0 mL/min using MeCN/0.1% acetic acid in H_2O 50/50, 80/20 as an eluent. Semi-preparative HPLC purification system was performed on an Agilent Eclipse XDB C18 column (5 µm, 9.6 × 250 mm) with the flow rate 5.0 mL/min using MeCN/0.1% acetic acid in H_2O 5/95 for 4 min, 5/95 to 45/55 gradient for 1 min and then 45/55 for 20 min. A dose calibrator (ATOMLAB 500, Biodex) was used for all radioactivity measurements.

Chemistry

4-amino-*N′*-hydroxy-*N*-(2-methoxyethyl)-1,2,5-oxadiazole-3-carboximidamide (2)

Compound 1 (4-amino-*N*-hydroxy-1,2,5-oxadiazole-3-carbimidoyl chloride) (3.99g, 24.6mmol) was disolved in ethyl acetate (20mL) and cooled in ice/water bath. Then, 2-methoxyethylamine (2.03g, 27.0mmol) was added dropwise followed by addation of triethylamine (5.14mL, 36.9mmol). The precipitation was observed after addation of triethylamine. After stirring 5min in the ice/water bath, the completeness of the reaction was confirmed by TLC. The organic mixture was washed by water (30mL), brine (20mL) and dried over Na_2SO_4. After evaporate the solvent, the crude brown liquid was obtained (5.1g, 103%). Small portion of crude product (~ 0.2g) was purified by flash chromatography (SiO_2) and eluted with ethyl acetate/Hexanes (2:3, v/v) to afford pure comound 2 as light yellow liquid for analytical purpose.

Rf (ethyl acetate/hexanes: 2/3 (v/v)): 0.20.

1H-NMR (400 MHz, $CDCl_3$): δ 5.70(br, 2 H, NH2), 5.35(s, 1H, NH), 3.78(t, J=4.8 Hz, 2H, CH2), 3.56(t, J=4.8 Hz, 2H, CH2) and 3.39 (s, 3H, OCH3).

13C-NMR (100 MHz, $CDCl_3$): δ 155.0, 146.0, 139.4, 72.4, 58.8, 43.6.

HRMS Calcd for C6H12N5O3 $[M + H]^+$, 202.0935, found, 202.0932.

N′-hydroxy-4-((2-methoxyethyl)amino)-1,2,5-oxadiazole-3-carboximidamide (3)

The crude compound 2 (5.10g) was mixed with water (20mL) and potassium hydroxide (4.13g, 73.7mmol). The reaction mixture was refluxed at 100°C overnight and the completeness of the reaction was confirmed by TLC. The reaction mixture was extracted by ethyl acetate (20mL × 3) and the combined organic layer was washed by brine (20mL) and dried over Na_2SO_4. After evaporate the solvent, the yellow oil was obtained as crude product 3 (5.10g, 100%).

Rf (ethyl acetate/hexanes: 2/3(v/v) w/ 1% NH_4OH): 0.18.

1H-NMR (400 MHz, $CDCl_3$): δ 8.58 (br, 1H, OH), 6.01(t, J=6.0Hz, 1H NH), 5.34(br, 2 H, NH2), 3.64(t, J=4.8Hz, 2H, CH2), 3.52(t, J=4.8Hz, 2H, CH2) and 3.39(s, 3H, OCH3).

13C-NMR (100 MHz, $CDCl_3$): δ 154.9, 144.6, 139.1, 70.4, 58.8, 43.7.

HRMS Calcd for C6H12N5O3 $[M + H]^+$, 202.0935, found, 202.0935.

N-hydroxy-4-((2-methoxyethyl)amino)-1,2,5-oxadiazole-3-carbimidoyl chloride (4)

The crude compound 3 (4.2g) was mixed with concentrated HCl (13.9mL), water (25mL), ethyl acetate (25mL), NaCl (3.68g, 63.0mmol) at the ice/water bath. Then $NaNO_2$ (1.45g, 21.0mmol in 10mL water) was added slowly. The reaction mixture was stirred under ice/water bath for 2 h and then at R.T. overnight. The yellow crude mixture was extracted by ethyl acetate (50mL × 2) and the combined organic layer was washed by brine (50mL) and dried over Na_2SO_4. After evaporate the solvent, the white solid was obtained as crude product 4 (4.63 g, 100%).

Rf (ethyl acetate/hexanes: 1/2 (v/v)): 0.20.

1H-NMR (400 MHz, $CDCl_3$): δ 9.93(br, 1H, OH), 3.69(t, J=4.8Hz, 2H, CH2), 3.56(t, J=4.8Hz, 2H, CH2) and 3.44(s, 3H, OCH3).

13C-NMR (100 MHz, $CDCl_3$): δ 154.2, 140.9, 130.8, 70.3, 58.9, 43.7.

N-(3-chloro-4-fluorophenyl)-*N'*-hydroxy-4-((2-methoxyethyl)amino)-1,2,5-oxadiazole-3-carboximidamide (5)

The crude compound 4 (4.63g) was mixed with water (30mL) and 3-chloro-4-fluoroaniline (3.06g, 21.0mmol). The reaction mixtue was stirred at 60°C for 10 min folleded by slowly added $NaHCO_3$(2.65g, 31.5mmol in 20mL water). Then the reaction was stirred at 60°C for 20min. After leave the reaction mixture stirred in R.T. for overnight, the completeness of the reaction was confirmed by TLC. The crude mixture was extracted by ethyl acetate (50mL× 2) and the combined organic layer was dried over Na_2SO_4. After evaporate the solvent, the brown solid was purified by flash chromatography (SiO_2) and eluted with ethyl acetate/Hexanes (1:1, v/v) to afford pure comound 5 as light yellow solid (5.22 g, 76%).

Rf (ethyl acetate/hexanes: 1/2(v/v)): 0.20.

1H-NMR (400 MHz, *d6*-DMSO): δ 11.58 (s, 1H, OH), 8.91(s, 1H, NH), 7.21(dd, J= 9.2 and 9.2Hz, 1H, Ar-H), 7.00(dd, J= 6.4 and 2.8Hz, 1H, Ar-H), 6.76(ddd, J = 8.8, 4.0 and 2.8Hz, 1H, Ar-H), 6.16(t, J = 6.0Hz, 1H NH), 3.51(t, J = 5.6Hz, 2H, CH2), 3.38(t, J = 5.6Hz, 2H, CH2) and 3.28 (s, 3H, OCH3).

13C-NMR (100 MHz, *d6*-DMSO): δ 155.7, 152.7(d, J^1_{C-F} =239.4Hz), 139.8, 139.5 137.8, 122.2, 120.9 (d, J^3_{C-F} =7.0 Hz), 118.6 (d, J^2_{C-F} =18.5Hz), 116.2(d, J^2_{C-F} =21.7Hz), 144.6, 139.1, 69.7, 58.1, 43.5.

HRMS Calcd for C12H13ClFN5O3Na $[M + Na]^+$, 352.0583, found, 352.0593.

4-(3-chloro-4-fluorophenyl)-3-(4-((2-methoxyethyl)amino)-1,2,5-oxadiazol-3-yl)-1,2,4-oxadiazol-5(4H)-one (6)

The crude compound 5 (5.22 g) was disolved in ethyl aceteate (50 mL) and then 1,1'-carbonyldiimidazole (3.88g, 23.9mmol) was added. After stirred at 60°C for 20min, the completeness of the reaction was confirmed by TLC. Then the reaction mixture was washed by 1N HCl (2×50mL) and dried over Na_2SO_4. the crude prodcut was purified by flash chromatography (SiO_2) and eluted with ethyl acetate/Hexanes (1:4, v/v) to afford compound 6 as off white solid (5.30 g, 94%).

Rf (ethyl acetate/hexanes: 1/4(v/v)): 0.19.

1H-NMR (400 MHz, $CDCl_3$): δ 7.50(ddd, J=6.0, 1.6 and 0.8Hz, 1H, Ar-H), 7.31(dd, J=0.8 and 0.8Hz, 1H, Ar-H), 7.30(d, J=1.6 Hz, 1H, Ar-H), 5.77(t, J=5.2 Hz, 1H NH), 3.62(t, J=4.8Hz, 2H, CH2), 3.55 (t, J=5.2Hz, 2H, CH2) and 3.40 (s, 3H, OCH3).

13C-NMR (100 MHz, $CDCl_3$): δ 160.4, 157.2(d, J^1_{C-F}=131.9Hz), 155.5, 148.9, 132.8, 130.3, 127.9(d, J^3_{C-F} =8.1Hz), 127.2(d, J^3_{C-F}=4.0Hz), 122.4(d, J^2_{C-F}=9.3Hz), 117.6(d, J^2_{C-F}=22.7Hz), 69.8, 58.9, 44.3.

HRMS Calcd for C13H12ClFN5O4 $[M + H]^+$, 356.0556, found, 356.0551.

4-(3-chloro-4-fluorophenyl)-3-(4-((2-hydroxyethyl)amino)-1,2,5-oxadiazol-3-yl)-1,2,4-oxadiazol-5(4H)-one (7)

Compound 6 (3.93g, 11mmol) was dissolved in dichloromethane (30 mL) and cooled by dry ice/acetone bath. Then, boron tribromide (2.09mL, 22mmol) was added dropwise at -78°C. The reaction mixture was warmed to -10°C for 30min and then R.T. for 1h. The completeness of the reaction was confirmed by TLC. The reaction mixture was quenched by $NaHCO_3$ sat. solution (75 mL) under ice/water bath. The crude mixture was extracted by ethyl acetate (25mL× 2) and the combined organic layer was dried over Na_2SO_4. After evaporated the solvent, the crude brown solid was purified by flash chromatography (SiO_2) and eluted with ethyl acetate/Hexanes (1:2, v/v) to afford pure comound 7 as white solid (3.11g, 82%).

Rf (ethyl acetate/hexanes: 1/2(v/v)): 0.20.

1H-NMR (400 MHz, *d6*-DMSO): δ 8.00(dd, J=6.4 and 2.4Hz, 1H, Ar-H), 7.70(ddd, J=8.8, 4.4 and 2.4Hz, 1H, Ar-H), 7.63(dd, J=8.8 and 8.8 Hz, 1H, Ar-H), 6.35(t, J=5.6 Hz, 1H, NH), 4.89(s, br, 1H, OH), 3.59(t, J=5.6Hz, 2H, CH2) and 3.31 (t, J=5.6Hz, 2H, CH2).

13C-NMR (100 MHz, *d6*-DMSO): δ 159.4, 156.8, 156.8(d, J^1_{C-F}=108.5Hz), 149.0, 133.8, 130.7, 129.5 (d, J^3_{C-F} =8.3Hz), 128.4 (d, J^3_{C-F} =3.6Hz), 120.1 (d, J^2_{C-F} =19.1Hz), 117.8(d, J^2_{C-F}=22.5Hz), 59.5, 46.5.

HRMS Calcd for C12H9ClFN5O4 $[M + H]^+$, 342.0400, found, 342.0403.

4-(3-chloro-4-fluorophenyl)-3-(4-((2-fluoroethyl)amino)-1,2,5-oxadiazol-3-yl)-1,2,4-oxadiazol-5(4H)-one (8)

Compound **7**(0.342g, 1.0mmol) was dissolved in dichloromethane (5mL) and Dimethylaminosulfur trifluoride(0.39mL, 4.0mmol) was added dropwise under argon protection. After stirred at 40 °C overnight, the completeness of the reaction was confirmed by TLC. Ethyl acetate (15mL) was added and then the reaction was quenched by $NaHCO_3$ Sat. solution (20mL) at 0°C. The organic layer was washed with $NaHCO_3$ Sat. solution (20mL×2), Brine (20mL) and dried over Na_2SO_4. After evaporated the solvent, the crude product was purified by flash chromatography (SiO_2) and eluted with ethyl acetate/Hexanes (1:3, v/v) to afford pure comound 8 as white solid (0.28 g, 81%).

Rf (ethyl acetate/hexanes: 1/3(v/v)): 0.24.

1H-NMR (400 MHz, $CDCl_3$): δ), 7.50(ddd, J=6.4, 2.0 and 0.4Hz, 1H, Ar-H), 7.26-7.35(m, 2H, Ar-H), 5.61(s, br, 1H, OH), 4.65(dd, J=47.6 and 4.8Hz, 2H, CH2), 3.72(ddd, J =24.4, 9.6 and 4.8Hz, 2H, CH2).

13C-NMR (100 MHz, $CDCl_3$): δ 160.6, 158.9, 155.9(d, J^1_{C-F} =120.7Hz), 149.0, 132.9, 130.4, 128.0 (d, J^3_{C-F} =8.1Hz), 127.2 (d, J^3_{C-F} =3.6Hz), 122.6 (d, J^2_{C-F} =19.1Hz), 117.8(d, J^2_{C-F} =22.7 Hz), 81.0(d, J^1_{C-F} = 167.3Hz), 45.0 (d, J^2_{C-F} = 20.4Hz),.

HRMS Calcd for C12H9ClF2N5O3 $[M + H]^+$, 344.0357, found, 344.0356.

2-((4-(*N*-(3-chloro-4-fluorophenyl)-*N′*-hydroxycarbamimidoyl)-1,2,5-oxadiazol-3-yl)amino)ethyl 4-methylbenzenesulfonate (9, Precursor)

To a solution of compound **7** (2.08g, 6.09mmol, in 20mL dichloromethan) was slowly added 4-Toluenesulfonyl chloride (1.28g, 6.70mmol) and triethylamine (1.7mL, 12.2mmol) under the ice/water bath. The reaction mixture was stirred 5min at 0°C then overnight at R.T. The completeness of the reaction was confirmed by TLC. After add water (20mL), the organic layer was collected and thhe water layer was then extraced by ethyl aceteat (10mL × 2). The combined organic layer was then washed by brine (20mL) and dried over Na_2SO_4. After evaporated the solvent, the crude product was purified by flash chromatography (SiO_2) and eluted with ethyl acetate/Hexanes (1:2, v/v) to afford pure comound 9 as white solid (2.0g, 66%).

Rf (ethyl acetate/hexanes: 1/2(v/v)): 0.29.

1H-NMR (400 MHz, *d6*-DMSO): δ, 8.02(dd, J=6.8 and 2.8Hz, 1H, Ar-H), 7.71 (m, J=8.4Hz, 3H, Ar-H), 7.66(dd, J=8.8 and 8.8Hz, 1H, Ar-H), 7.39(d, J=8.4Hz, 2H, Ar-H), 6.67(t, J=5.6Hz, 1H, NH), 4.18(t, J=5.2Hz, 2H, CH2), 3.49(dd, J=10.8 and 5.2Hz, 2H, CH2), 2.33(s, 3H, CH3).

13C-NMR (100 MHz, *d6*-DMSO): δ 159.4, 156.8(d, J^2_{C-F} =12.3Hz), 154.8, 148.7, 145.0, 133.7, 131.0(d, J^1_{C-F} =94.1Hz), 130.6, 129.6 (d, J^3_{C-F} =8.2Hz), 128.4 (d, J^3_{C-F} =3.6Hz), 127.7, 120.2 (d, J^2_{C-F} = 19.0Hz), 117.8(d, J^2_{C-F} =22.5Hz), 66.9, 42.9, 21.0.

HRMS Calcd for C19H16ClFN5O6S $[M + H]^+$, 496.0488, found, 496.0477.

N-(3-chloro-4-fluorophenyl)-4-((2-fluoroethyl)amino)-*N′*-hydroxy-1,2,5-oxadiazole-3-carboximidamide (IDO49)

To a solution of compound 8 (0.69g, 2.00mmol, in 16 mL tetrahydrofuran) was added sodium hydroxide solution(2N, 4.0mL) and stirred at R.T. 1h. The completeness of the reaction was confirmed by TLC. After evaporated the tetrahydrofuran, the reaction mixture was extracted by ethyl acetate (10mL × 2). The combined organic layer was then washed by brine (20mL) and dried over Na_2SO_4. After evaporated the solvent, the crude product was purified by flash chromatography (SiO_2) and eluted with ethyl acetate/Hexanes (1:4, v/v) to afford pure comound IDO49 as white solid (0.62 g, 98%).

Rf (ethyl acetate/hexanes: 1/4(v/v)): 0.10.

1H-NMR (400 MHz, *d6*-DMSO): δ11.56(s, 1H, OH), 8.93(s, 1H, NH), 7.21(dd, J=8.8 and 8.8Hz, 1H, Ar-H), 7.00(dd, J=6.4 and 2.4Hz, 1H, Ar-H), 6.76(ddd, J=8.8, 4.40 and 2.8Hz, 1H, Ar-H), 6.36(t, J=5.6Hz, 1H, NH), 4.60(dd, J=47.6 and 4.8Hz, 2H, CH2) and 3.56(ddd, J = 27.2, 10.4 and 5.2, Hz, 2H, CH2).

13C-NMR (100 MHz, *d6*-DMSO): δ 155.6, 154.0, 151.6, 139.9, 138.6(d, J^1_{C-F}=161.0Hz), 122.3, 121.0 (J^3_{C-F} =6.9Hz), 118.7(d, J^2_{C-F} =18.6Hz), 116.2(d, J^2_{C-F} =21.8Hz), 81.6 (d, J^1_{C-F} =163.9Hz), 44.4(d, J^2_{C-F} =20.1Hz).

HRMS Calcd. for C11H11ClF2N5O2 $[M + H]^+$, 318.0564, found, 318.0554.

REFERENCE

1. Huang X, Gillies RJ, Tian H. Synthesis of [(18) F] 4-amino-N-(3-chloro-4-fluorophenyl)-N'-hydroxy-1,2,5-oxadiazole-3-carboximidamide (IDO5L): a novel potential PET probe for imaging of IDO1 expression. J Labelled Comp Radiopharm. 2015; 58:156-162.

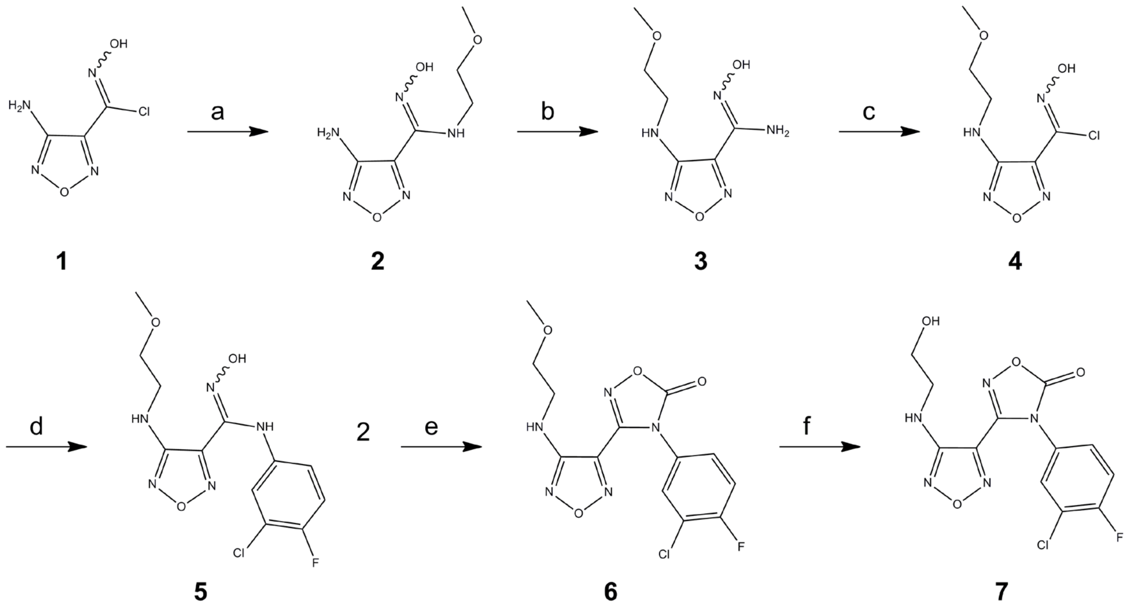

Supplementary Scheme 1: Synthesis of compound 7. a. 2-methoxyethylamine, ethyl acetate, Et3N, 0°C 5 min; b. KOH, water, 100°C, overnight; c. $NaNO_2$, NaCl, HCl (aq.), 0°C 2h then R.T. overnight; d. water, 3-chloro-4-fluoroaniline, $NaHCO_3$, 60°C 20 min then R.T. overnight. 4-step overall yield 64% ; e. 1,1'-carbonyl diimidazole, ethyl acetate, 60°C 20 min, 94%. f. BBr3, dichloromethane, -78°C to -10°C 30 min, then R.T. 1h, quenched by $NaHCO_3$ saturated solution R.T. 10 min, 82%.
